# Investigation of the Efficacy of Epidermal Growth Factor Receptor (EGFR)-Tyrosine Kinase Inhibitor in Patients With EGFR Exon 21 L858R Point Mutation-Positive Non-small Cell Lung Cancer

**DOI:** 10.7759/cureus.64811

**Published:** 2024-07-18

**Authors:** Yutaka Takahara, Ryudai Abe, Sumito Nagae, Takuya Tanaka, Yoko Ishige, Ikuyo Shionoya, Kouichi Yamamura, Masafumi Nojiri, Masaharu Iguchi

**Affiliations:** 1 Respiratory Medicine, Kanazawa Medical University, Uchinada, JPN

**Keywords:** pdl1 expression, interstitial lung disease, nsclc, egfr gene mutation, egfr exon 21 l858r point mutation, egfr-tki

## Abstract

Background: Treatment of advanced non-small cell lung cancer (NSCLC) with epidermal growth factor receptor (EGFR)-tyrosine kinase inhibitors (TKIs) has a higher response rate than with conventional chemotherapy in patients positive for EGFR mutations. However, the efficacy of EGFR-TKI therapy may be reduced in patients positive for the EGFR exon 21 L858R point mutation.

Objective: To determine the clinical characteristics of patients with EGFR exon 21 L858R point mutation-positive NSCLC who are non-responders to EGFR-TKI therapy and the factors that predict response to EGFR-TKI therapy.

Methods: Patients with NSCLC treated with EGFR-TKIs were evaluated for response after treatment, and those who responded were compared with those who did not respond.

Results: Of 31 patients, 21 (67.7%) responded to EGFR-TKI therapy (the response group). There were significantly more programmed death ligand 1 (PDL1)-negative patients in the response group than in the non-response group. A significantly higher number of patients in the PDL1-positive group developed interstitial lung disease (ILD) after EGFR-TKI therapy than those in the PDL1-negative group.

Conclusion: EGFR-TKI therapy is likely to be non-responsive in PDL1-positive patients with EGFR exon 21 L858R point mutation-positive NSCLC. The PDL1-positive group is at a high risk of developing ILD.

## Introduction

Epidermal growth factor receptor (EGFR)-tyrosine kinase inhibitors (TKIs) have significantly improved response rates compared with conventional chemotherapy in patients with EGFR mutations in non-small cell lung cancer (NSCLC) [1-4]. Therefore, EGFR-TKIs are widely used as first-line systemic therapy for patients with unresectable advanced or metastatic EGFR-mutated NSCLC [5,6]. EGFR exon 19 deletions or EGFR exon 21 L858R point mutations are commonly referred to as EGFR mutations because they account for approximately 90% of EGFR mutations and are EGFR-TKI sensitive mutations [7].

However, 10-30% of patients with positive EGFR mutations do not benefit from EGFR-TKI therapy and may even experience rapid disease progression [1-4].

In addition, EGFR-TKI therapy has been suggested to have a lower efficacy for patients with the exon 21 L858R point mutation than for patients with exon 19 deletion [8,9]. Moreover, some reports suggest that co-mutation of tumor suppressor genes may be responsible for the differential response to EGFR-TKI therapy [10]; however, the exact mechanism remains unclear.

To date, no study has exclusively targeted EGFR exon 21 L858R point mutation-positive NSCLC. Identifying the subpopulations of patients with exon 21 L858R point mutations who are unlikely to benefit from EGFR-TKI therapy may lead to the development of personalized medicine and optimal treatment strategies.

Therefore, the purpose of this study was to determine the clinical characteristics of patients with EGFR exon 21 L858R point mutation-positive NSCLC who do not respond to EGFR-TKI therapy and the factors that predict response to EGFR-TKI therapy. The study compared EGFR-TKI-treated patients with EGFR exon 21 L858R point mutation-positive NSCLC who achieved a response with those who did not.

## Materials and methods

**Figure 1:**
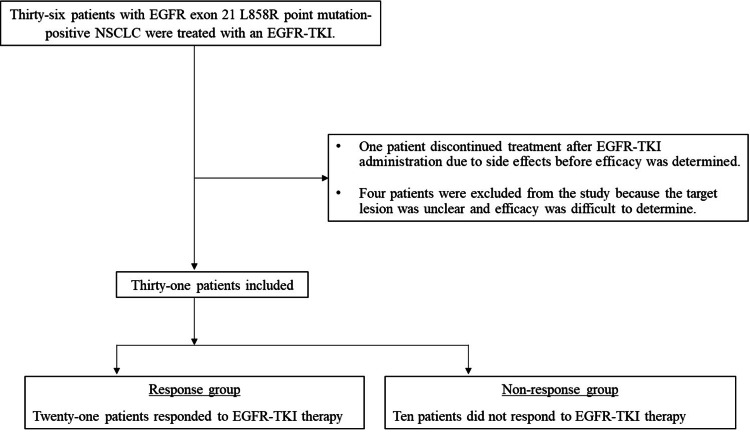
Study flowchart. Thirty-six patients with EGFR exon 21 L858R point mutation-positive NSCLC treated with an EGFR-TKI were included in the study. Of these, one patient whose treatment was discontinued due to side effects after EGFR-TKI administration before efficacy was determined and four patients with indeterminate target lesions were excluded. Ultimately, 31 patients were included in the study.

Patients with EGFR exon 21 L858R point mutation-positive NSCLC who were treated with EGFR-TKIs for advanced-stage (stage IIIB, IV, postoperative relapse) NSCLC between July 2012 and December 2023 were retrospectively selected. Patients whose treatment was terminated owing to side effects after EGFR-TKI administration before efficacy was determined and patients with unclear target lesions were excluded from the study because it was difficult to assess the response rate. Patient characteristics are depicted in Figure 1.

Data on age, sex, smoking history, performance status (PS), histological type of lung cancer, tumor proportion score, treatment details, and side effects at the time of initial EGFR-TKI treatment were collected.

Responses were evaluated according to the Response Evaluation Criteria in Solid Tumors, version 1.1. Patients with complete and partial responses were classified as the response group, and patients with stable disease and progressive disease were classified as the non-response group; the clinical information of the two groups was compared.

The severity of interstitial lung disease (ILD) was assessed using the Common Terminology Criteria for Adverse Events, version 5.0.

Treatment protocol

Patients were treated with an EGFR-TKI, including gefitinib 250 mg/day, erlotinib 150 mg/day, afatinib 40 mg/day, or osimertinib 80 mg/day. Treatment was administered until disease progression or unacceptable toxicity. Drug dose reductions were based on physician judgment.

Immunohistochemical staining

Immunohistochemistry (IHC) staining was performed in all cases using the PDL1 kit (PDL1 IHC 22C3 pharmDX; Dako) according to the manufacturer's instructions [11].

Analysis of EGFR gene mutations

Tissue samples obtained from primary tumors or metastatic lesions were analyzed using Cycleave-PCR for testing prior to 2021. After 2022, next-generation sequencing (NGS) analysis was performed using the Oncomine Dx Target Test (Ion Torrent PGM Dx Sequencer; Thermo Fisher Scientific, Norristown, PA).

In this study, EGFR mutations were not detected using cytology samples. These samples were submitted to SRL Laboratories, a private Japanese laboratory, for testing.

Statistical analysis

All analyses were performed using Statistical Product and Service Solutions (SPSS, version 26.0; IBM SPSS Statistics for Windows, Armonk, NY). Two-sided p-values of <0.05 were considered statistically significant. All categorical variables were analyzed using the chi-square test, except for variables with a predicted frequency of <5, which were analyzed using Fisher’s exact probability test. We used t-tests without correspondence for the mean comparisons of continuous variables between the two groups.

Survival curves were generated using the Kaplan-Meier method, and the analysis covered the period from the start of lung cancer treatment to death or termination of treatment. Survival analysis was conducted in mid-April 2024. The log-rank test was used to analyze whether there was a difference in survival due to differences in response to EGFR-TKI administration.

## Results

A total of 31 cases were identified. All patients were Japanese with lung adenocarcinomas and received EGFR monotherapy. None of the patients concomitantly received platinum-based agents or angiogenic inhibitors. EGFR monotherapy included osimertinib in 16 patients, gefitinib in eight patients, erlotinib in six patients, and afatinib in one patient.

Regarding the evaluation of the response in the overall population, 21 patients (67.7%) had partial response, eight (25.8%) had stable disease, and two (6.5%) had progressive disease. Thus, 21 patients (67.7%) were in the response group, and 10 (32.3%) were in the non-response group. The patient characteristics are shown in Table 1.

**Table 1:** Comparison of patient characteristics between the response and non-response groups. †Based on Fisher’s exact test. Abbreviations: ECOG, Eastern Cooperative Oncology Group; PS, performance status; PDL1, programmed death ligand 1; ICI treatment, immune checkpoint inhibitor treatment after tyrosine kinase inhibitor treatment; ILD, interstitial lung disease; EGFR-TKI, epidermal growth factor receptor-tyrosine kinase inhibitor; TKI, tyrosine kinase inhibitors

	Response group	Non-response group	p-value
n (%)	21 (67.7%)	10 (32.3%)	
Age (years), mean (range)	71.7 (48–85)	74.2 (59–86)	0.470
Sex (male/female)	8/13	2/8	0.428
Smoking history (never/prior, current)	8/13	4/6	0.919
ECOG PS (0–1/2–4)	19/2	9/1	1.000
Clinical stages (IV, postoperative recurrence/III)	19/2	9/1	1.000
Size of the target lesion, mean (range)	37.8 (11.0–81.0)	33.7 (14.0–51.0)	0.501
Distant metastasis (yes/no)			
Brain metastasis	7/14	2/8	0.677
Pleural metastasis	9/12	5/5	0.595
PDL1 expression (22C3) (%), (0/1-100/ untested)	9/5/7	0/4/6	0.049^†^
TKI treatment duration (month), mean (range)	12.4 (0.4–66.2)	7.4 (0.4–21.3)	0.223
Treatment setting for TKI (first/second line, or later)	20/1	9/1	1.000
ICI treatment (yes/no)	2/19	2/8	0.577
EGFR-TKI rechallenge (yes/no)	13/8	3/7	0.508
ILD (yes/no)	4/17	5/5	0.076
Osimertinib (yes/no)	10/11	6/4	0.398

Programmed death ligand 1 (PDL1)-negative patients were significantly more common in the response group than in the non-response group (p=0.049). There was also a trend toward a higher number of patients with ILD in the non-response group than in the response group (p=0.076). There was no significant difference in the use of osimertinib between the two groups. There were no significant differences in age, sex, smoking history, PS, clinical stage, or timing of treatment between the two groups.

The types of adverse events that required dose reduction or discontinuation after EGFR-TKI administration are shown in the appendix (Supplement Table 4). The only intolerable adverse event with gefitinib was ILD (25.0%). The most common unacceptable adverse event with erlotinib was skin/mucositis (50.0%), followed by ILD (16.7%). The most common adverse event with osimertinib was ILD (37.5%), followed by liver dysfunction (12.5%) (Appendix Supplement Table 1).

The backgrounds of PDL1-positive patients (the PDL1-positive group) and PDL1-negative patients (the PDL1-negative group) are shown in Table 2. Nine PDL1-positive (50%) and nine PDL1-negative (50%) patients were identified. The PDL1-positive group was dominated by non-smokers (p=0.048).

**Table 2:** Comparison of patient characteristics between the PDL1-positive and PDL1-negative groups. *p<0.05. †Based on Fisher’s exact test. Abbreviations: ECOG, Eastern Cooperative Oncology Group; PS, performance status; PDL1, programmed death ligand 1; ICIs treatment, immune-checkpoint inhibitor treatment after tyrosine kinase inhibitor treatment; ILD, interstitial lung disease; EGFR-TKI, epidermal growth factor receptor-tyrosine kinase inhibitor; TKI, tyrosine kinase inhibitor

	PDL1-positive	PDL1-negative	p-value
n (%)	9 (50%)	9 (50%)	
Age (years), mean (range)	68.6 (59–79)	66.3 (29–77)	0.525
Sex (male/female)	4/5	4/5	0.233
Smoking history (never/prior, current)	5/4	3/6	0.048*
ECOG PS (0–1/2–4)	9/0	7/2	0.267
Clinical stages (IV, postoperative recurrence/III)	9/0	7/2	0.267
Size of the target lesion, mean (range)	40.8 (17.6–64.9)	41.5 (18.6–81.0)	0.934
Distant metastasis (yes/no)			
Brain metastasis	2/7	4/5	0.481
Pleural metastasis	2/7	3/6	0.200
TKI treatment duration (month), mean (range)	6.04 (0.4–13.8)	15.4 (0.9–66.2)	0.216
Treatment setting for TKI (first /second line, or later)	0/9	1/8	0.613
ICI treatment (yes/no)	3/6	0/9	0.082
EGFR-TKI rechallenge (yes/no)	5/4	4/5	0.876
ILD (yes/no)	6/3	0/9	0.006^†^
Osimertinib (yes/no)	7/2	5/4	0.091

A higher number of patients in the PDL1-positive group developed ILD than those in the PDL1-negative group (p=0.006). There were no significant differences in age, sex, PS, clinical stage, or treatment timing between the two groups.

The clinical characteristics, treatments, and side effects in the PDL1-positive group are shown in Table 3.

**Table 3:** Patient details for the PDL1-positive group. Abbreviations: TPS, tumor proportion score; evaluation, initial response evaluation after epidermal growth factor receptor-tyrosine kinase inhibitor treatment; ICIs, immune checkpoint inhibitor treatment after epidermal growth factor receptor-tyrosine kinase inhibitor therapy; discontinuance reason, reasons for discontinuation of epidermal growth factor receptor-tyrosine kinase inhibitor therapy; M, male; F, female; ILD, interstitial lung disease; PD, progressive disease; NE, not valuable; BOR, best overall response; TKI, tyrosine kinase inhibitor

Case (age/sex)	PS	TPS (%)	Evaluation	TKI (Treatment Phase)	Duration of TKI (Month)	Discontinuation Reason	ILD (Grade)	ICI (Duration (Cycle)/BOR)	TKI Rechallenge (Duration (Month)/BOR)
1 (74/M)	1	50	SD	Osimertinib (first-line)	1.2	ILD	5	None	None
2 (72/M)	0	1	PR	Osimertinib (first-line)	0.4	ILD	4	None	3^rd^; Gefitinib (28.7/SD)
3 (59/F)	0	10	SD	Osimertinib (first-line)	13.8	PD	None	None	None
4 (64/F)	1	15	PR	Erlotinib (first-line)	4.5	ILD	2	5^th^; Atezolizumab (3/SD)	None
5 (79/F)	1	20	PR	Osimertinib (first-line)	2.9	ILD	2	None	None
6 (62/M)	0	30	SD	Osimertinib (first-line)	9.6	PD	None	5^th^; Atezolizumab (3/ PD)	3^rd^; Gefitinib (9.5/PR)
7 (77/M)	0	40	PR	Erlotinib (first-line)	11.2	PD	None	3^rd^; ABCP (1/NE)	2^nd-^, Osimertinib (26.2/PR)
8 (75/F)	1	1	SD	Osimertinib (first-line)	0.5	ILD	1	None	3^rd^; Gefitinib (0.4/NE)
9 (48/F)	1	55	PR	Osimertinib (first-line)	10.3	On-going	None	None	None

**Figure 2:**
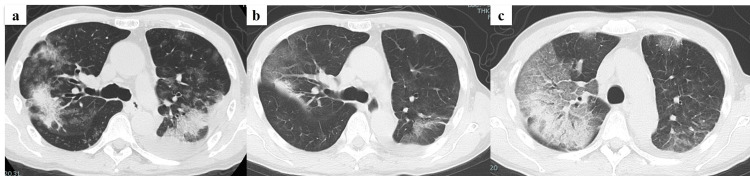
Computed tomography image of the chest. A 72-year-old male patient with lung adenocarcinoma presented with multiple lung metastases and pleurisy (Case 2). Osimertinib monotherapy was initially administered. On the 11th day of treatment, fever and respiratory failure were observed, and chest computed tomography (CT) was performed. A CT scan of the chest revealed that the metastatic lung tumor had shrunk. However, a newly developed, extensive frosted shadow was observed, mainly in the right upper lobe. Drug-induced lung injury was diagnosed, and osimertinib was discontinued. (a) Chest CT before the initiation of epidermal growth factor receptor-tyrosine kinase inhibitor therapy; (b) chest CT at interstitial lung disease onset

**Figure 3:**
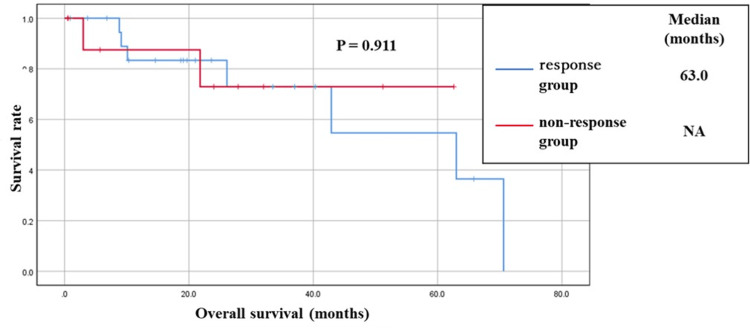
Overall survival in the response and non-response groups. The median survival time in the response group was 63.0 months, whereas that in the non-response group was NA. There was no significant difference between the two groups (p=0.911, log-rank test). NA, not applicable

All patients received an EGFR-TKI as the first-line therapy. Seven of the nine patients (77.8%) were initially treated with osimertinib. Five of the nine patients (55.6%) developed ILD, and one patient died of ILD exacerbation. TKI therapy was discontinued in all five patients who developed ILD. The representative imaging findings of a patient with ILD in the PDL1-positive group are shown in Figure 2. The survival curves for the response and non-response groups are shown in Figure 3. There was no significant difference between the two groups (p=0.911, log-rank test).

## Discussion

Patients with EGFR mutation-positive lung cancer who are non-responders to first-line EGFR-TKI therapy have been suggested to have decreased overall survival (OS) compared with responders [12]. When planning optimal treatment strategies for EGFR exon 21 L858R point mutation-positive patients, it is important to determine the clinical characteristics of EGFR-TKI non-responders.

In this study, there were significantly more PDL1-negative patients in the response group than in the non-response group. High PD-L1 expression has been reported to be associated with a poor prognosis in patients with positive EGFR mutations [13-17]. The results of this study suggest that PDL1-positive patients with EGFR exon 21 L858R point mutations may have a reduced response to EGFR-TKI therapy, which may be associated with poor prognoses. However, there has been a report that PDL1 negativity is associated with decreased OS in patients positive for EGFR exon 21 L858R point mutation [18]. In this study, there was a case in which a patient was PDL1-positive but achieved a good response to lung metastases as early as eight days after EGFR-TKI therapy (Case 2 in Table 3). It has also been reported that PDL1 expression status is not associated with OS in EGFR mutation-positive patients [19]. Thus, consistent results linking PD-L1 expression and EGFR mutations to prognosis have not been demonstrated [9].

Previous reports have included all subtypes of the EGFR gene, and further investigation is needed with a large sample size of only EGFR exon 21 L858R point mutation-positive patients to determine whether positive PDL1 expression is associated with a decreased response to EGFR-TKI therapy in patients with EGFR exon 21 L858R point mutation. Herein, a significantly higher number of ILD cases were observed in the PDL1-positive group than in the PDL1-negative group. ILD development during EGFR-TKI therapy is associated with treatment discontinuation and shortened prognosis [20,21]. Eight of 31 patients (25.8%) had to discontinue treatment because of the development of ILD, and three patients (9.7%) died owing to worsening ILD. In the PDL1-positive group, a high rate of ILD development was observed in five of eight patients (63%). Thus, EGFR exon 21 L858R point mutation-positive patients with PDL1 positivity may be at a high risk of developing ILD when using TKIs. These results suggest that PDL1-positive patients with EGFR exon 21 L858R point mutation positivity may require special attention to prevent the development of ILD when on EGFR-TKI therapy.

In this study, PDL1-positive patients who were at a high risk of developing ILD were more common in the non-response group than in the response group. ILD tended to occur more frequently in the non-response group than in the response group. Based on these results, one would expect a poor prognosis in the non-response group; however, there was no difference in prognosis between the two groups. A reason for the lack of a difference in prognosis between the two groups may be that there was no difference in the duration of EGFR-TKI therapy between them. In EGFR-TKI therapy for EGFR mutation-positive patients, it has been suggested that there is a survival benefit to administering a TKI if the patient remains clinically stable after radiological tumor progression [22]. In the present study, many patients in the non-response group were able to maintain their disease status for a long period after the initial treatment, even if they did not achieve a response.

Another reason could be the impact of cancer treatment after EGFR-TKI therapy. In patients without the T790M mutation after first- or second-generation EGFR-TKI treatment failure and after third-generation EGFR-TKI treatment failure, there are reports of rechallenge with an EGFR TKI as a treatment option, with some efficacy being demonstrated [23-25]. In this study, there was no significant difference in the number of patients who were introduced to the EGFR-TKI rechallenge between the response and non-response groups. However, reports of EGFR-TKI rechallenge have shown varied results, with a median progression-free survival of 2.0-8.0 months and an overall response rate of 7-25% [25]. The possibility that there were many non-responders in whom the EGFR-TKI rechallenge was effective cannot be ruled out. Future evaluation of the efficacy of EGFR-TKI rechallenges in patients with EGFR exon 21 L858R point mutation positivity is needed.

This study has several limitations. First, this was a single-center retrospective study, which had the potential for bias in patient selection and data collection. However, the findings of this study may help identify the optimal patient population of EGFR exon 21 L858R point mutation-positive NSCLC patients who would benefit from EGFR-TKI therapy, in turn improving the prognosis of patients with NSCLC. Future studies should include large multicenter studies to mitigate this shortcoming. Second, the treatment protocol was not clearly defined, and the type of TKI therapy used in the initial treatment was left to the discretion of the attending physician rather than being uniformly applied, which could have led to bias among the study groups.

Transient asymptomatic pulmonary opacity has been reported in approximately 35% of patients during osimertinib treatment [26,27]. In addition, a higher frequency of ILD has been reported with osimertinib than with first- and second-generation EGFR-TKIs in Japanese patients [28-30]. Nonetheless, herein, there was no difference in osimertinib therapy between the two groups even though all patients were Japanese. Further studies with large sample sizes are needed to determine whether the obtained results of ILD incidence in this study can be generalized.

## Conclusions

The findings of this study suggest that PDL1-positive patients with the EGFR exon 21 L858R point mutation are potentially less responsive to EGFR-TKI therapy. Identifying the characteristics of patients with this mutation who may not benefit from EGFR-TKI therapy can help with the development of personalized medicine and optimal treatment strategies. In particular, our findings indicate that PDL1-positive patients with EGFR exon 21 L858R point mutations may require close monitoring to prevent the development of ILD when using EGFR-TKI therapy. Thus, we hope our study will raise awareness and inspire future research, thereby leading to better patient outcomes in this population.

## Appendices

**Table 4:** Types of adverse events in patients administered an EGFR-TKI. Abbreviations: EGFR-TKI, epidermal growth factor receptor-tyrosine kinase inhibitor; ILD, interstitial lung disease

Adverse event	Gefitinib (n=8)	Erlotinib (n=6)	Osimertinib (n=16)
Skin/Mucositis	0	3 (50.0)	1 (6.3)
ILD	2 (25.0)	1 (16.7)	6 (37.5)
Liver dysfunction	0	0	2 (12.5)
Neutropenia	0	0	1 (6.3)
